# The Corrosion Fatigue Behavior and Mechanism of AerMet 100 Steel in 3.5% NaCl at Room Temperature

**DOI:** 10.3390/ma17205025

**Published:** 2024-10-14

**Authors:** Donghua Tian, Can Xu, Hongli Wang, Chengchuan Wu, Yonghao Lu

**Affiliations:** 1 National Center for Materials Service Safety, University of Science and Technology Beijing, Beijing 100083, China; tdh1015@ustb.edu.cn (D.T.);; 2 Chengdu Advanced Metal Materials Industrial Technology Institute Co., Ltd., Chengdu 610300, China

**Keywords:** corrosion fatigue, fatigue life, AerMet 100 steel, crack growth, crack initiation

## Abstract

AerMet 100 steel is a new type of double-hardened high-strength steel, which is often used as landing gear material in amphibious aircraft. In the present paper, the corrosion fatigue behavior and mechanism of AerMet 100 high-strength steel in a 3.5% NaCl solution was studied by stress-controlled fatigue tests and a series of subsequent characterizations of the fracture surface, microstructure, and cracks. The results indicated that the fatigue life of AerMet 100 high-strength steel decreased with a decrease in the stress level in a 3.5% NaCl solution, satisfying the relationship lgN = −2.69 × 10^−3^ σ + 6.49. The corrosion fatigue crack usually initiated from the corrosion pit and propagated across the martensitic flat noodles. Meanwhile, the corrosion fatigue crack tip was filled with Cr_2_O_3_, Fe_2_O_3_, and amorphous material; it propagated in the transgranular mode by a slip dissolution mechanism. This study provides some engineering significant for the fatigue performance of AerMet 100 steel in a 3.5% NaCl solution.

## 1. Introduction

It is impossible to avoid the impact of stress corrosion, corrosion fatigue, and other factors which result in material failure and cracking under specific environmental service conditions that lead to serious catastrophic accidents [1,2,3]. Therefore, in engineering material application design, the factors contributing to material failure should be concerned and cannot be underestimated [4,5,6]. AerMet 100 steel is a new type of secondary hardening high-strength steel that has a high hardness, strength, and excellent ductility and toughness [7,8,9]. At the same time, due to its excellent resistance to stress corrosion, cracking and fatigue, it is widely used in important structural components in applications such as aircraft landing gear [6,10]. Currently, research has shown that the microstructural characteristics and mechanical properties of AerMet 100 high-strength steel are related to its strength, mainly due to the precipitation of M_2_C during the annealing process, and its fracture toughness, mainly caused by the thickness of the austenitic layer during the annealing process [11,12]. Shi et al. [13] studied the influence of microstructures at a tempering temperature near 482 °C on the mechanical properties of AerMet 100 high-strength steel, showing that the comprehensive mechanical properties of AerMet 100 at 482 °C were the best; tempering at a temperature higher than 482 °C leads to the growth of M_2_C, and an increase in inverse martensitic film, which affects its toughness. Wang et al. [14,15] studied the effects of the structure of M_2_C precipitates at different times on its mechanical properties at 482 °C. The results showed that M_2_C would coarsen with the prolongation of the duration of tempering, and that M_2_C mainly existed in needle-like, sheet-like, and rod-shaped structures, leading to a decrease in its mechanical properties. Zhang et al. [16] reported that cryogenic treatment effectively reduced the content of austenite. Nanophase M_2_C was precipitated in a martensite structure, and austenite was mainly distributed at the boundary of martensite flat noodles, playing a role in strengthening and toughening. Hu et al. [17] studied the hydrogen embrittlement phenomenon in AerMet 100 steel during slow strain rate tensile testing (SSRT) using the hydrogen permeation method. The results showed that in acidic or aqueous solutions, the main reason for the decrease in its performance was the occurrence of hydrogen permeation. M_2_C, as a hydrogen trap, hindered the diffusion of hydrogen inside. Donahue et al. [18] investigated the interaction between corrosion fatigue and stress corrosion cracking in precipitation-hardened martensitic stainless steel in chloride environments and the corrosion mechanism at the crack’s tip and the influence of hydrogen. Figueroa et al. [7] found that in slow strain rate tensile testing (SSRT), transgranular fractures in AerMet 100 steel typically extend from multiple lateral points toward the center. Previous studies on E690 steel or other steels indicate that crack initiation is greatly promoted without the formation of pits and cracks and do not initiate at pits, even though pits are already produced.

Up until now, it has been crucial to clarify the corrosion fatigue life, crack initiation, and crack propagation mechanism of AerMet 100 steel in a seawater environment. To determine this, in this study, the complex service environment of amphibious aircraft landing gear was simulated, the corrosion fatigue life curve was identified, and the mechanism of crack initiation and crack propagation during corrosion fatigue was clarified.

## 2. Materials and Experimental Methods

The AerMet 100 high-strength steel used in this experiment was produced by a domestic enterprise at 482 °C for 5 h. The content of O, N, and H elements in AerMet 100 high-strength steel was determined using an ONH-2000 oxygen, nitrogen, and hydrogen analyzer. The content of C and S elements were determined using an ELTRA CS800 carbon sulfur analyzer, while the content of other major elements was measured using ICP-OES. The specific chemical composition of AerMet 100 high-strength steel is shown in Table 1.

**Figure 1:**
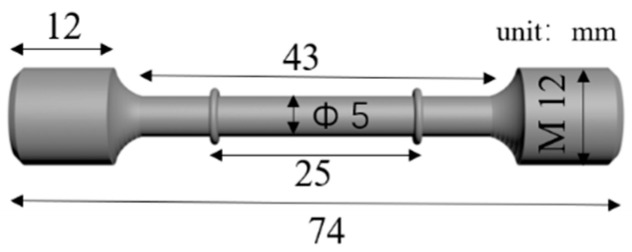
Tensile sample geometry of the SSRT specimen in a 3.5 wt.% NaCl solution.

The slow strain rate tensile test specimen was prepared, as shown in Figure 1, according to the following standard [19]: GB/T 15970.7-2017 Corrosion Stress Corrosion Testing of Metals and Alloys Part 7: Slow Strain Rate Test.

**Figure 2:**
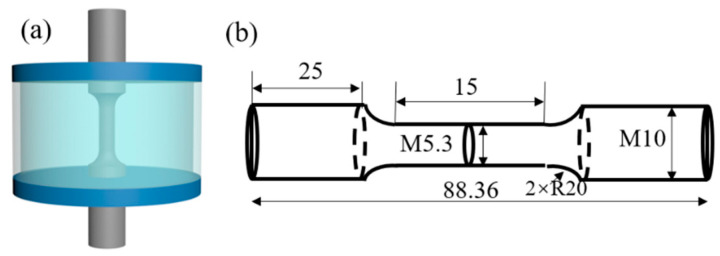
The schematic diagram (**a**) and geometry (**b**) of the AerMet 100 steel corrosion fatigue specimen in which the dimension unit is mm.

In order to study the stress corrosion sensitivity of AerMet 100 high-strength steel in a 3.5% NaCl environment, slow strain rate tensile experiments were conducted at a selected strain rate of 6.67 × 10^−7^ s^−1^ at room temperature and in a 3.5% NaCl solution environment, respectively. Fatigue experiments were carried out according to the following relevant standard [20]: Method for Axial Force Control in Metal Material Fatigue Experiments (GB/T 3075-2021). Smooth round bar specimens were used for fatigue performance testing. The test schematic is shown in Figure 2. The surface of the cut sample was first polished to a metallographic state, and corrosion fatigue performance testing was conducted using an electro-hydraulic servo fatigue machine. Five sets of fatigue life tests were conducted at room temperature without cyclic stress levels (979 MPa, 579 MPa, 479 MPa, 379 MPa, and 279 MPa, respectively), with three parallel samples tested in each set. The stress ratio of cyclic stress is −1, and the loading waveform is a sine wave with a loading frequency of 1 Hz.

After finishing the corrosion fatigue performance testing, the extracted samples were washed with clean water and subjected to ultrasonic oscillation with an alcohol solution for 10 min to remove surface contaminants. Then, the samples were vacuum-dried and stored for analysis. The surface and port morphology of the sample were analyzed using Oxford Instruments (SEM, Aztec X-MAXN 50, Carl Zeiss, Oberkochen, Germany) field emission scanning electron microscopy and EBSD analysis using Oxford Instruments (Nordlys Max3, Oberkochen, Germany). Surface corrosion products were analyzed for phase by TTRII18kW Cu target X-ray diffraction. Characterization of oxide crystal structure of corrosion fatigue crack tip used FEI Talos F200X field emission transmission electron microscopy (TEM, Talos F200X, Bend, OR, USA). The microcracks on the side were cut along the axial direction.

## 3. Results

**Figure 3:**
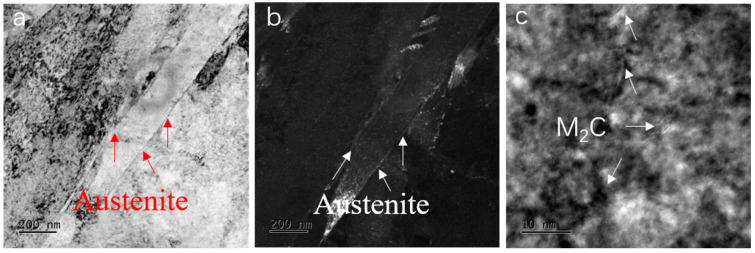
The HRTEM image of AerMet 100 steel tempered for 5 h; (**a**) bright field image; (**b**) dark field image; (**c**) high-resolution image of M_2_C.

Figure 3 shows a TEM image of the microstructure of AerMet 100 high-strength steel tempered at 482 °C for 5 h. Figure 3a–c shows the bright-field images, inverted austenite diffraction spots and dark field images of the microstructure of AerMet 100 high-strength steel tempered for 5 h, respectively. It can be observed in the bright-field images (Figure 3a) and dark-field images (Figure 3b) that a large number of precipitates were distributed inside the martensitic flat noodles, and reverse austenite films were distributed near the flat noodle boundary. In addition, acicular carbide (M_2_C) can be observed at a high resolution in Figure 3c.

**Figure 4:**
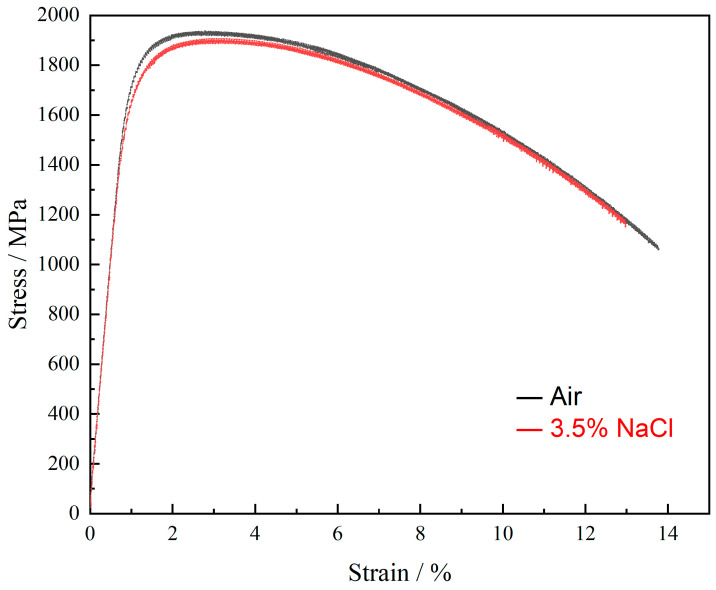
Stress–strain behavior of AerMet 100 steel at different conditions (black: air, and red: 3.5% NaCl, strain rate: 6.67 × 10^−7^ s^−1^ s^−1^).

Figure 4 presents the engineering stress–strain curves for AerMet 100 high-strength steel tempered for 5 h in air and a 3.5 wt.% NaCl environment at room temperature, respectively. It can be clearly seen from Figure 4 that the strength in the atmospheric environment was higher than that in the 3.5% NaCl solution, and its strain lasted for longer.

The stress corrosion susceptibility of AerMet 100 high-strength steel in 3.5% NaCl solution was evaluated using the elongation rate δ of the sample and the cross-sectional shrinkage rate ψ of the fracture surface. The specific calculation formulae are as follows:

$$\delta =\frac{I_{1}-I_{2}}{I_{1}}\times 100\%$$

$$\psi =\frac{A_{1}-A_{2}}{A_{1}}\times 100\%$$

$$I_{\delta }=(1-\frac{\delta_{s}}{\delta_{O}})\times 100\%$$

$$I_{\psi }=(1-\frac{\psi_{s}}{\psi_{O}})\times 100\%$$

In the formulae, I_1_ represents the original gauge length of the slow strain rate tensile specimen; I_2_ represents the gauge length of the slow strain rate tensile specimen after completing the test; A_1_ represents the original cross-sectional area of the gauge length section of the slow strain rate tensile specimen of AerMet 100 high-strength steel; A_2_ represents the cross-sectional area of the slow strain rate tensile specimen of AerMet 100 high-strength steel after the end of the test; *δ*_s_ represents the elongation of the AerMet 100 high-strength steel sample in a 3.5% NaCl solution; *δ*_o_ represents the elongation of AerMet 100 high-strength steel specimens in air; I_δ_ represents the elongation loss rate of the AerMet 100 high-strength steel samples in a 3.5% NaCl solution; *ψ*_s_ represents the cross-sectional shrinkage rate of AerMet 100 high-strength steel samples in a 3.5% NaCl solution; *ψ*_o_ represents the cross-sectional shrinkage rate of AerMet 100 high-strength steel specimens in air; I*_ψ_* represents the cross-sectional shrinkage loss rate of AerMet 100 high-strength steel samples in a 3.5% NaCl solution.

Table 2 presents the yield strength, tensile strength, *δ*_s_, I*_δ_*, *ψ*_s_, and I*_ψ_* of AerMet 100 high-strength steel. The yield strength and tensile strength of the specimens in air were 1754 MPa and 1930 MPa, respectively, which were significantly higher than those in 3.5% NaCl solution at 1661 MPa and 1803 MPa. Additionally, the *δ*_s_ and *ψ*_s_ of the specimens decreased, indicating an increase in the brittleness of AerMet 100 high-strength steel during the slow strain rate tensile testing in the solution.

**Figure 5:**
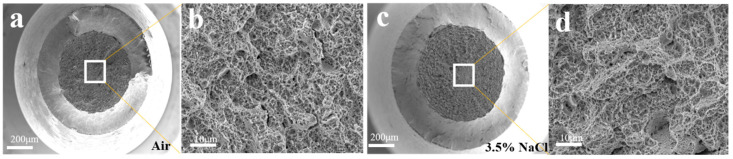
The fracture morphology of AerMet 100 steel in different environments after SSRTs: (**b**) and (**d**) are the partially magnified images of the corresponding zones in (**a**) air and (**c**) in 3.5% NaCl solution, respectively.

Figure 5 presents SEM images of the fracture morphologies of AerMet 100 high-strength steel after slow strain rate tensile testing at room temperature atmosphere and 3.5% NaCl solution, respectively. From the macroscopic fracture morphology, it can be seen that both of the fracture morphologies were composed of a central fiber zone and a shear lip zone. Under the conditions of room temperature atmosphere and a 3.5% NaCl solution environment, the samples exhibited the necking phenomenon, indicating that the material still had certain toughness characteristics when it fractured. However, as the environment changed from a room temperature environment to a simulated seawater environment, the necking phenomenon gradually weakened, the section shrinkage rate showed a decreasing trend, and the brittleness increased. And compared with the tensile section of the material in air and environmental media, the fracture surface under the simulated seawater environmental media conditions was smoother and the shrinkage rate of the fracture surface was lower. From the microstructure, it can be seen that in the air environment (Figure 5b), the magnified microstructure of the fracture surface of the sample showed, essentially, a dimple morphology. The fracture morphology in the 3.5% NaCl solution exhibited ductile fracture characteristics (Figure 5d), with a locally magnified microscopic morphology consisting of secondary cracks and ductile dimples with cleavage and quasi cleavage morphologies.

**Figure 6:**
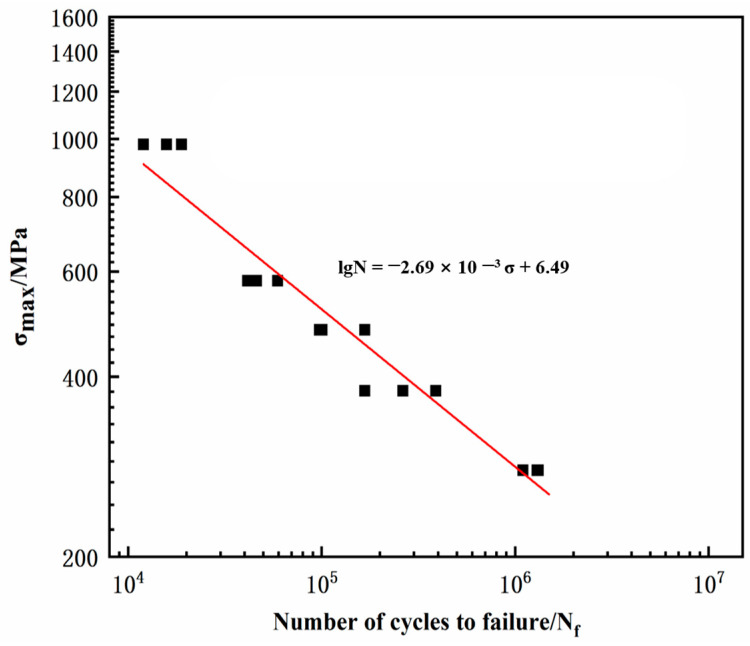
Maximum stress versus number of cycles to failure.

After corrosion fatigue tests, a scatter plot was obtained with the experimental cycle number N as the horizontal axis and stress level S as the vertical axis, and the fatigue life S-N curve was fitted. Figure 6 shows the fatigue life curve of AerMet 100 high-strength steel in a 3.5% NaCl solution environment. As shown in Figure 6, the cyclic life of the AerMet 100 high-strength steel gradually increased with the decrease in five different stress levels (979 MPa, 579 MPa, 479 MPa, 379 MPa, and 279 MPa, respectively). The fatigue life data in the 3.5% NaCl solution was fitted(red line) using the Weibull formula, which is expressed as follows:

lgN = −2.69 × 10^−3^ σ + 6.49

Obviously, the fatigue life of the AerMet 100 high-strength steel gradually decreased with the decrease in stress level, and the fatigue life was negatively correlated with the stress level in a 3.5% NaCl solution environment, showing that the AerMet 100 high-strength steel had no fatigue limit.

**Figure 7:**
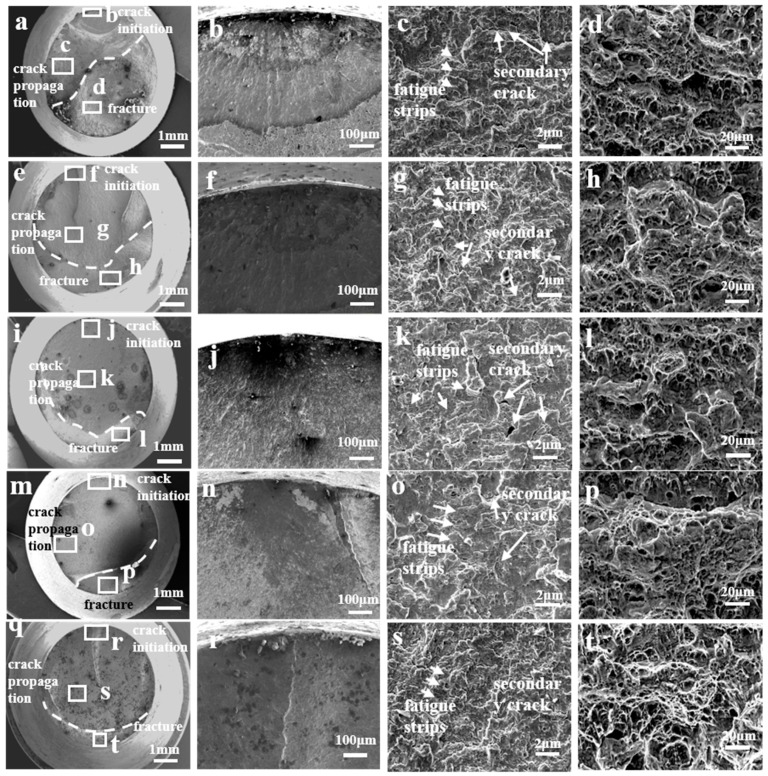
The fracture morphology of AerMet 100 high-strength steel with corrosion fatigue under different stresses ((**a**): 979 MPa, (**e**): 579 MPa, (**i**): 479 MPa, (**m**): 379 MPa, and (**q**): 279 MPa, respectively) in a 3.5% NaCl solution; (**b**–**d**) magnified image corresponding to (**a**), (**f**–**h**) magnified image corresponding to (**e**), (**j**–**l**) magnified image corresponding to (**i**), (**n**–**p**) magnified image corresponding to (**m**), (**r**–**t**) magnified image corresponding to (**q**), respectively.

Figure 7 presents SEM images of the fatigue fractographies of the AerMet 100 high-strength steel in a 3.5% NaCl solution at different stress levels (979 MPa, 579 MPa, 479 MPa, 379 MPa, and 279 MPa, respectively). It can be observed that the three regions appeared in the fractographies at different stress levels: crack source region, crack propagation region, and instantaneous fracture region. The fracture surface of the fatigue specimen showed an obvious necking phenomenon, and the fatigue fracture surface in the 3.5% NaCl solution became smoother with a decrease in the stress level. In order to further observe the initiation and propagation of the cracks, an enlarged image of the crack source (in Figure 7) showed that, in a 3.5% NaCl solution, different degrees of transgranular fracture occurred near the crack source at different stress levels. The proportion of transgranular fracture increased with the decrease in the applied stress level, and the farther away from the crack source, the more obvious the transgranular fracture characteristics, indicating that the 3.5% NaCl solution caused the fatigue crack initiation and propagation in the AerMet 100 steel to transfer from the intragranular to the grain boundary. There were obvious fatigue strips and secondary cracks in the crack propagation zone, and a large number of toughness dimples and secondary cracks appeared in the instantaneous fracture zone.

**Figure 8:**
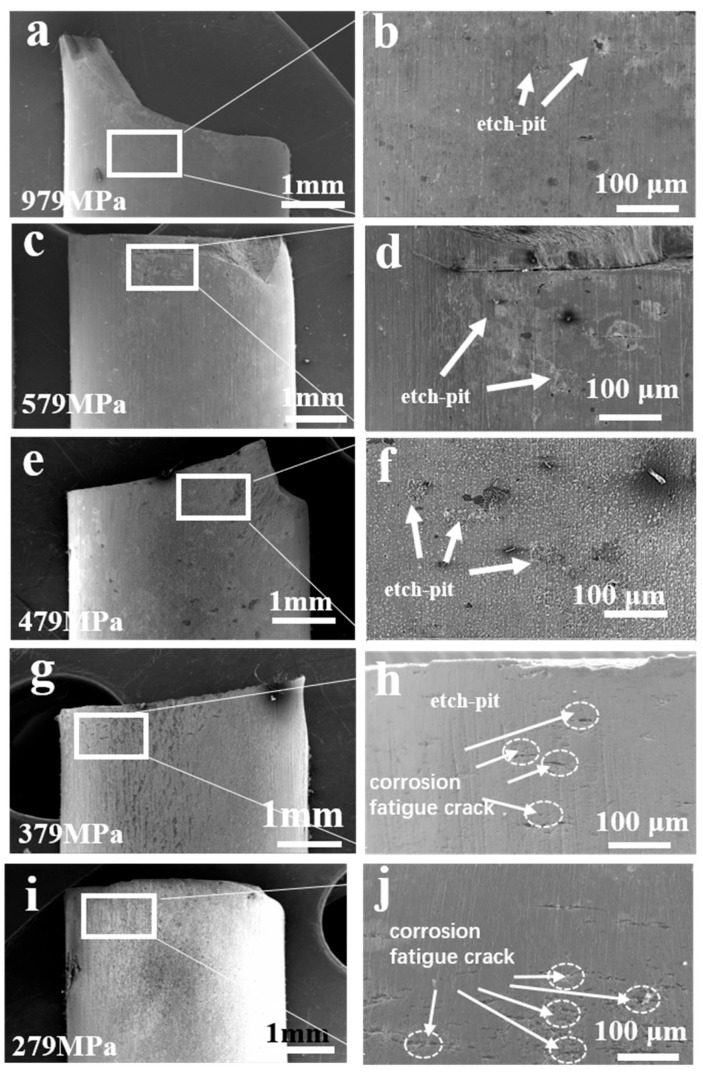
The external image of AerMet 100 high-strength steel with corrosion fatigue under different stresses ((**a**): 979 MPa, (**c**): 579 MPa, (**e**): 479 MPa, (**g**): 379 MPa, and (**i**): 279 MPa; (**b**,**d**,**f**,**h**,**j**) were magnified image corresponding to (**a**,**c**,**e**,**g**,**i**), respectively) in a 3.5% NaCl solution.

Figure 8 presents SEM images of the lateral morphology of the fatigue specimens of the AerMet 100 high-strength steel under different stresses in a 3.5% NaCl solution. It was found that, in the 3.5% NaCl solution, only corrosion pits rather than microcracks were clearly observed in the fatigue specimens at high stress levels (979 MPa, 579 MPa, and 479 MPa, respectively). By comparison, at low stress levels (379 MPa and 279 MPa), a large number of corrosion pits and obvious microcracks at the bottom of the corrosion pits could be clearly observed on the side of the fatigue specimens, and the corrosion fatigue cracks were perpendicular to the stress loading direction, indicating that microcracks initiated at the corrosion pits under low stress in the 3.5% NaCl solution environment.

**Figure 9:**
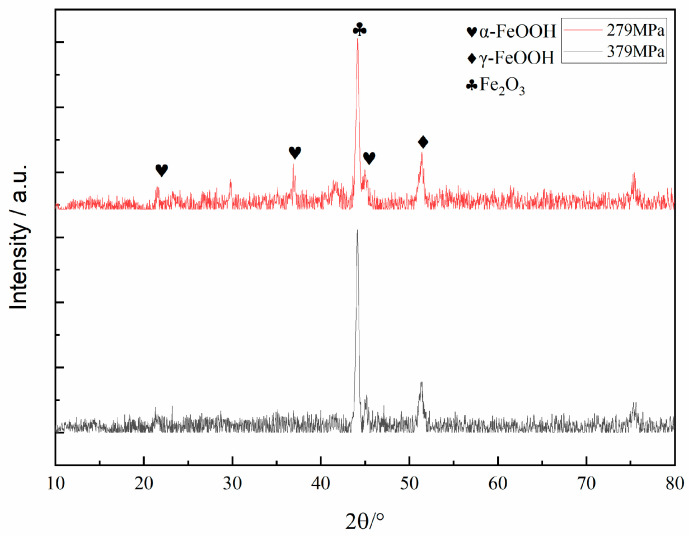
The XRD patterns of the surface corrosion products of corrosion fatigue specimens of AerMet 100 steel under different stresses (279 MPa, 379 MPa) in a 3.5% NaCl solution.

Figure 9 presents XRD patterns of the surface corrosion products of the corrosion fatigue specimens of AerMet 100 steel under different stresses (279 MPa, 379 MPa) in a 3.5% NaCl solution. It was found that the corrosion products generated during the corrosion fatigue test in the 3.5% NaCl solution were mainly Fe_2_O_3_ and FeOOH. 

**Figure 10:**
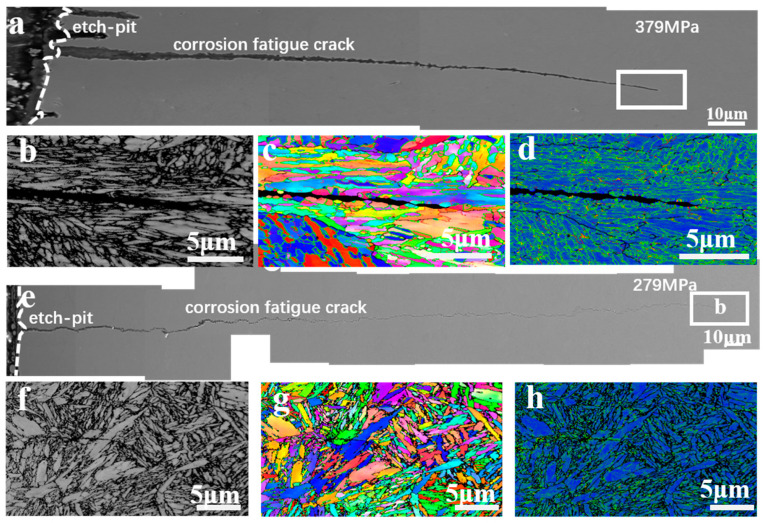
The SEM and EBSD analysis of the cross-section corrosion products of corrosion fatigue specimens of AerMet 100 steel under different stresses (379 MPa images were in (**a**–**d**), 279 MPa images were in (**e**–**h**)) in 3.5% NaCl solution.

Figure 10 shows the cross-sectional maps of the fatigue microcracks under 379 MPa (Figure 10a–d) and 279 MPa (Figure 10e–h) in a 3.5% NaCl solution, respectively. It was found that the corrosion fatigue cracks usually originated from pitting corrosion pits and propagated into the matrix in Figure 10a,e. An EBSD analysis in Figure 10c,g showed that the corrosion fatigue crack propagates through the martensitic flat noodles transgranularly, and there was an obvious stress concentration near the crack tip.

**Figure 11:**
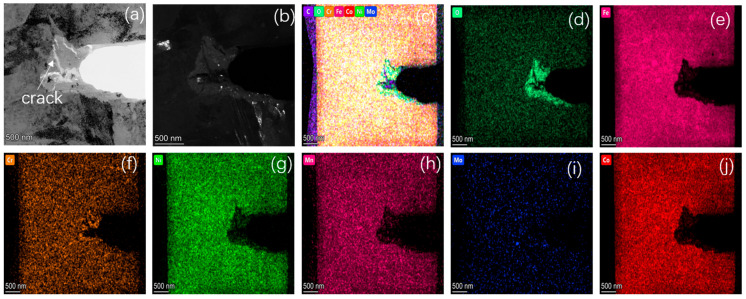
TEM and mapping of corrosion crack tip of AerMet 100 steel at 379 MPa in a 3.5 wt.%NaCl solution. (**a**) Bright-field image; (**b**) dark-field image; (**c**) multiple element maps are overlay; (**d**–**j**) the element maps of O, Fe, Cr, Ni, Mn, Mo, and Co, respectively.

**Figure 12:**
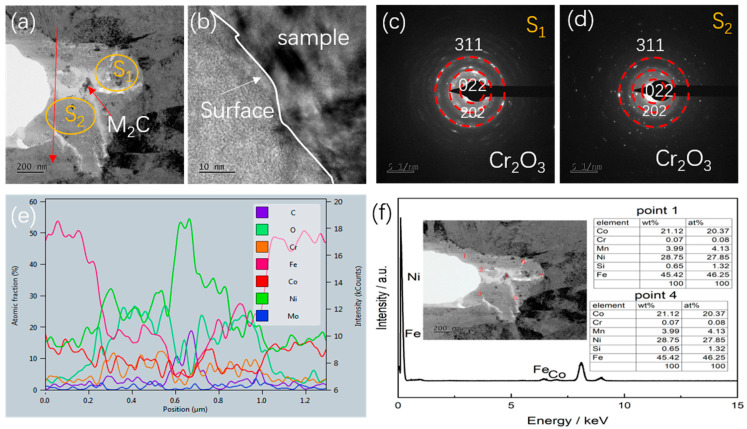
HRTEM and EDS diagram of corrosion crack tip of AerMet 100 steel at 379 MPa in a 3.5 wt.% NaCl solution. (**a**) Bright-field image; (**b**) HRTEM image of crack tip; (**c**,**d**) magnified images of the S1 and S2 regions, respectively; (**e**) the line scan pattern of the red line; and (**f**) the EDS results of point 1 and 4.

In order to clarify the propagation mechanism of the fatigue crack tip, the FIB was used to prepare the TEM samples for the characterization of the crack tip area. Figure 11 presents the corresponding TEM and mapping images of the corrosion crack tip area of the AerMet 100 steel in a 3.5% NaCl solution at 379 MPa. It can be seen that the crack tip was highly blunt, and filled with some oxides at the tip. From the mapping images, it can be observed that there was a significant accumulation of oxygen in the tip region, which overlapped with iron (Fe), chromium (Cr), and some nickel (Ni). In order to characterize the composition of the oxide, TEM diffraction, line scan, and point scan analyses were performed on the corrosion fatigue tip region (Figure 12). It could be observed that there were no obvious lattice bands in the high-resolution diffraction pattern, as shown in Figure 12b. Selective zone diffraction analyses were conducted on the tip oxidation areas S1 and S2, and it was found that Cr_2_O_3_ with crystal planes of 202, 322, and 311 was mainly formed. At the same time, surface scanning and point scanning clearly indicated the accumulation of a large amount of oxygen and the presence of Cr_2_O_3_ and Fe_3_O_4_ in the corrosion fatigue tip area, which was consistent with the corrosion products shown in Figure 9.

**Figure 13:**
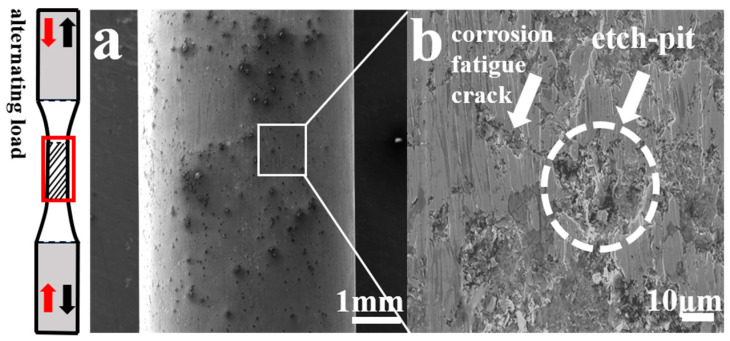
SEM image of interrupted sample of AerMet 100 steel at 279 MPa in 3.5% NaCl solution. (**a**) External morphology (from the red caption); (**b**) partially enlarged detail from figure (**a**).

In order to further investigate the mechanism of corrosion fatigue crack initiation and propagation, an interrupted sample of AerMet 100 high-strength steel at 279 MPa specimen with 60% N (fatigue life) in a 3.5% NaCl solution was studied. Figure 13 shows the lateral morphology of the sample. It can be observed that there are a large number of corrosion pits and secondary cracks distributed on the side of the interrupted sample, indicating that corrosion fatigue cracks already originated from the corrosion pits when the loading frequency of the corrosion fatigue sample reached 60% N (fatigue life).

**Figure 14:**
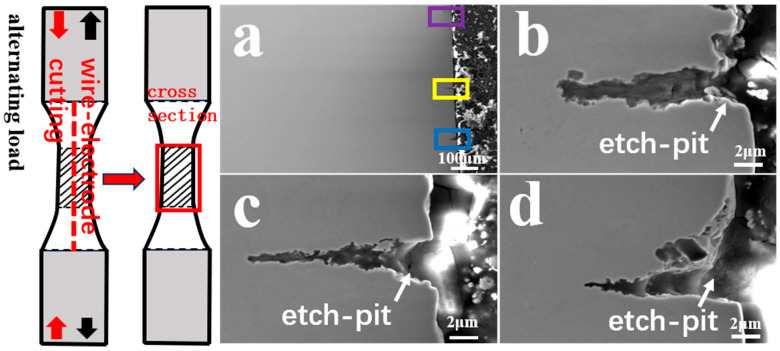
SEM image of crack growth with interrupted sample of AerMet 100 steel at 279 MPa in 3.5% NaCl solution. (**a**) Cross-sectional morphology(from the red caption); (**b**–**d**) the partially magnified images of the the purple, yellow and blue zones in (**a**), respectively.

In order to further investigate the corrosion fatigue crack propagation mechanism, the interrupted specimen of AerMet 100 steel at 279 MPa in a 3.5% NaCl solution was studied. Figure 14a presents the cross-sectional SEM morphology image of the corrosion fatigue interrupted specimen of AerMet 100 high-strength steel in a 3.5% NaCl solution. Some microcracks can be observed at the edge of the specimen section. Figure 14b–d are magnified images of the secondary cracks shown in Figure 14a. It could be observed that all the corrosion fatigue microcracks originated from the corrosion pits on the surface of the specimen and the corrosion fatigue cracks propagated forward under the action of applied stress.

**Figure 15:**
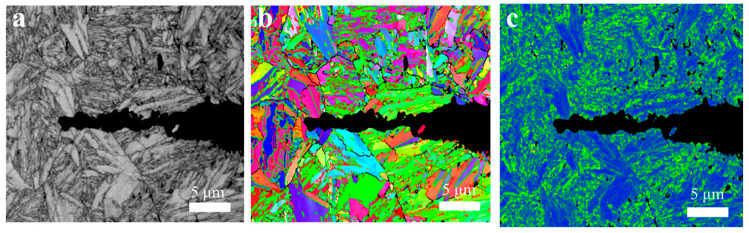
The (**a**) SEM, (**b**) IPF + GB, and (**c**) KAM image of crack growth with interrupted sample of AerMet 100 steel at 279 MPa in a 3.5% NaCl solution.

**Figure 16:**
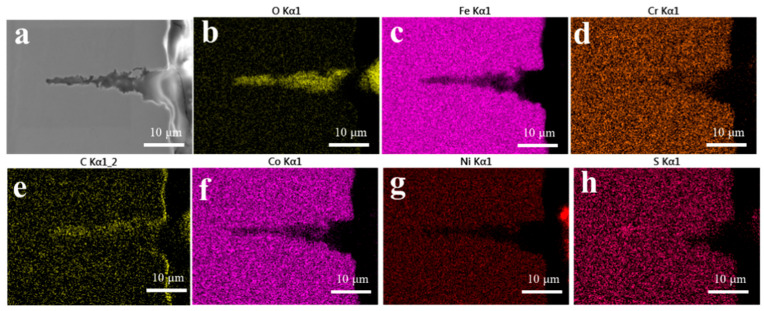
The EDS mapping image of (**a**) crack with interrupted sample of AerMet 100 steel at 279 MPa in 3.5% NaCl solution; (**b**–**h**) the element maps of O, Fe, Cr, C, Co, Ni, and Ss, respectively.

Figure 15 and Figure 16 show the EBSD analysis diagram (reverse pole diagram and grain boundary diagram (IPF + GB), local dislocation supporting role (KAM)) and EDS mapping image of one of the corrosion fatigue cracks in Figure 14. It was found that the crack propagated in the form of a transgranular fracture, which was consistent with the TEM observations in Figure 11 and Figure 12. The element distribution near the crack in Figure 11 shows that oxygen enrichment can be observed within the corrosion fatigue crack, indicating that oxides were mainly generated at the crack tip during the corrosion fatigue crack propagation. According to the HRTEM results in Figure 12, the oxide was believed to be mainly Cr_2_O_3_.

## 4. Discussion

Due to a series of water chemical reactions of AerMaet100 high-strength steel in a 3.5% NaCl solution, corrosion pits and cracks were formed, which led to the cracks expanding inward with the action of a load. The electrochemical behavior mainly included anode dissolution and cathodic oxygen absorption [21]. In a 3.5% NaCl solution, corrosion products might undergo the following reactions [22,23]:

Fe → Fe^2+^ + 2e^−^

Subsequently, Fe^2+^ participates in the hydrolysis reaction:

Fe^2+^ + 2H_2_O → Fe(OH)_2_ + 2H

In addition, Fe(OH)_2_ may undergo decomposition reactions:

2Fe(OH)_2_ → Fe_2_O_3_ + H_2_O + 2e^−^ + 2H^+^

As a result, galvanic differences between substances that are different from the collective took place, leading to anodic dissolution.

The fatigue life of AerMet 100 high-strength steel gradually decreased with the decrease in the stress level, and the fatigue life was negatively correlated with the stress level in the 3.5% NaCl solution environment, indicating that the AerMet 100 high-strength steel had no fatigue limit in a 3.5% NaCl solution environment. A similar conclusion was reached for E690 steel [22]. At the same time, prolonged immersion in NaCl solution would reduce the fatigue life of AerMet 100 high-strength steel, and the longer the immersion time in seawater, the longer the fatigue life. In a 3.5% NaCl solution environment, the fracture surface of the fatigue specimen of AerMet 100 high-strength steel showed three regions: a crack source zone, crack propagation zone, and instantaneous fracture zone [24]. The crack source in the fatigue specimens originated from the corrosion pits on the surface of the specimen, and expanded inside the matrix under the action of alternating stress. In the crack propagation zone, fatigue bands and secondary cracks could be observed, and the instantaneous fracture zone exhibited a dimple-like appearance. Moreover, in the 3.5% NaCl solution environment, a small number of corrosion pits but no obvious cracks on the side of the specimen could be clearly observed under high stress in the lateral morphology of the fatigue specimen of AerMet 100 high-strength steel, indicating that, under high stress, the fatigue life cycle was small, the immersion time in the solution was relatively short, and the effect of corrosion on the fatigue specimen was smaller, while the effect of mechanical fatigue on the specimen was greater. By comparison, corrosion pits and numerous cracks could be observed on the side of the specimen under low stress, and the cracks propagated inward from the corrosion pits, indicating that the corrosion fatigue cracks in the AerMet 100 high-strength steel originated from the corrosion pits in a 3.5% NaCl solution environment. The analysis of the sample cut along the axial direction through the corrosion pit showed that the cross-sectional cracks originated at the bottom of the corrosion pit, propagated forward under the action of stress concentration, and the corrosion fatigue crack propagation path exhibited transgranular characteristics. The microscopic analysis of the crack tip area showed that it propagated through the martensitic flat noodles, resulting in a large number of secondary cracks, and Cr_2_O_3_-based oxides were formed ahead of the crack tip. Meanwhile, anodic dissolution was observed at the crack tip. As shown in Figure 11, the microcrack initiated when the localized anodic dissolution reached a certain depth. During the initial propagation stage under low peak stress, the effects of the corrosion attack still dominated over the mechanical damage. As a result, this propagation mode under low peak stress (279 MPa) evolved into the transgranular fracture at the crack tip. This phenomenon was also found in the initial stage of stress corrosion crack formation in E690 steel [22].

## 5. Conclusions

In this study, the corrosion fatigue performance of AerMet 100 high-strength steel tempered at 482 °C for 5 h in 3.5% NaCl solution was comprehensively investigated, and the fatigue crack initiation and propagation mechanisms under different amounts of stress were analyzed in detail, and the influence of stress on the corrosion fatigue lifespan was evaluated. The major conclusions are as follows.

(1) The corrosion fatigue of AerMet 100 steel after heat treatment and tempering at 485 °C for 5 h gradually decreased with the decrease in stress level in a 3.5% NaCl solution at room temperature. The AerMet 100 high-strength steel had no fatigue limit on the stress level; the fatigue life was negatively correlated with stress, satisfying the relationship lgN = −2.69 × 10^−3^ σ + 6.49.
(2) In the 3.5% NaCl solution, the corrosion products were mainly Fe_2_O_3_ and FeOOH. The corrosion fatigue crack usually initiated from the corrosion pit and propagated across the martensitic flat noodles.
(3) The corrosion fatigue crack tip was filled with Cr_2_O_3_, Fe_2_O_3_, and amorphous material; it propagated in the form of a transgranular mode by the slip dissolution mechanism.

## Figures and Tables

**Table 1:** The chemical components of AerMet 100 steel (mass. %).

C	Co	Cr	Mo	Ni	P	Si	O	N	H
0.255	14.27	3.14	1.32	12.22	0.0041	0.155	0.0019	0.0009	0.00006

**Table 2:** Mechanical properties of AerMet 100 steel in air and 3.5% NaCl solution.

Atmosphere	Yield Strength (MPa)	Tensile Strength (MPa)	δ_s_ (%)	I_δ_ (%)	ψ_s_ (%)	I_ψ_ (%) s
Air	1754	1930	14.50	-	62.37	-
3.5% NaCl	1661	1903	13.20	8.96	60.79	2.5

## Data Availability

The raw/processed data required to reproduce these findings cannot be shared at this time as the data also form part of an ongoing study.
